# Association between dietary hardness score and activities of daily living among individuals aged 90 years

**DOI:** 10.1002/cre2.622

**Published:** 2022-06-24

**Authors:** Hidenori Urabe, Hiroshi Oue, Kyou Hiasa, Kazuhiro Tsuga

**Affiliations:** ^1^ Department of Dentistry Mitsugi General Hospital Onomichi Japan; ^2^ Department of Advanced Prosthodontics, Graduate School of Biomedical and Health Sciences Hiroshima University Hiroshima Japan

**Keywords:** ADL, dietary hardness score, older adults

## Abstract

**Objectives:**

With the rapidly aging world population, Japan has many older people with difficulties in maintaining oral health. This study aimed to investigate the relationship between dietary hardness score and performance of activities of daily living (ADL) of 90‐year‐old individuals in rural areas of Japan.

**Material and Methods:**

A total of 236 individuals (64 men and 172 women) aged 90 years in Mitsugi town, Hiroshima, Japan, were included. Assessment of oral status and survey of ADL were performed. The association of ADL with dietary hardness score and the number of remaining teeth was determined.

**Results:**

The mean dietary hardness score was 8.3 ± 2.6. Eighty individuals showed independence in ADL, whereas 156 individuals showed dependence. Logistic regression analysis, adjusted for potential confounding factors, showed that dietary hardness score was associated with ADL status but not the number of remaining teeth.

**Conclusion:**

Our findings suggest that a low dietary hardness score is associated with dependence on ADL in 90‐year‐old individuals.

## 1 INTRODUCTION

According to the United Nations estimates, the world centenarian population in 2015 was nearly half a million (Robine & Cubaynes, 2017). The Ministry of Health, Labour, and Welfare (MHLW: a cabinet‐level ministry of the Japanese government) in Japan reported that the centenarian population exceeded 80,000 for the first time in 2020, with Japan categorized as a super‐aged society (MHLW, 2020). The declining function of daily living and potential diseases are inevitable in older individuals. Factors contributing to centenarians' health are of interest to the scientific community (Bhardwaj et al., 2020). A previous study showed that a high percentage of centenarians remained functionally independent until age 90 (Hitt et al., 1999; Vetrano et al., 2021). Indeed, life expectancy is longer now than it was 50 years ago (Dicker et al., 2018). Extending healthy life expectancy is also important in maintaining the quality of life among older people.

Oral health affects overall health and life expectancy. Recent studies showed that functional dependency in activities of daily living (ADL) or instrumental ADL is associated with the oral health status of older people over the age of 60 years (de Lima Saintrain et al., 2021, 2018). A study investigating chewing self‐assessment and daily function revealed that chewing ability may be independently related to functional status in 80‐year‐old people (Takata, Ansai, Awano, Sonoki et al., 2004). Moreover, masticatory dysfunction may be an important risk factor for mortality in 80‐year‐old people (Nomura et al., 2020). These studies suggest a positive relationship between chewing ability or oral health and physical activity in older people. To our knowledge, however, there are few studies targeting individuals aged >90 years. Therefore, the present study aimed to assess the relationship between ADL and dietary hardness score, as an approximate measure of habitual chewing, in 90‐year‐old individuals.

## 2 MATERIALS AND METHODS

This study was approved by the Ethics Committee of Mitsugi General Hospital.

### 2.1 Participants

**Figure 1:**
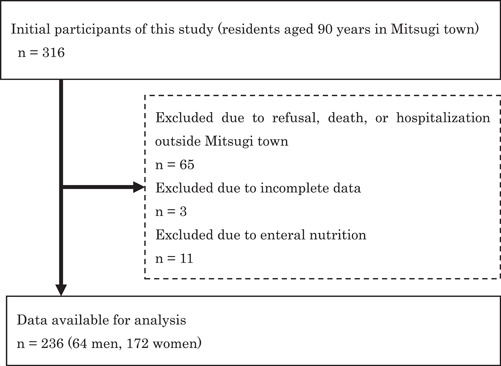
Flow chart of participant selection and study design.

We included all 90‐year‐old individuals in Mitsugi town, Hiroshima, Japan. Of the 316 initial participants, 236 were available for analysis, and their written consent was obtained (Figure 1). The survey was conducted for 5 years, from 2014 to 2018. We collected the data from individuals who turned 90 years old each year.

### 2.2 ADL assessment

The independent or dependent ADL were classified by participants who were certified to receive insurance for long‐term care (Supporting Information: Table S1) (Imahashi et al., 2007; Tsutsui & Muramatsu, 2005). We regarded as dependent ADL those who certified the following care levels; support required or care level 1, 2, 3, 4, and 5. Japanese universal health insurance coverage system covers all citizens with public medical care. So, most of the citizens 90‐year‐old in this city were included in this study. ADL status was identified from their medical records obtained from Mitsugi General Hospital or Mitsugi Health and Welfare Center.

### 2.3 Dietary hardness score

Dietary hardness score was evaluated using an estimate of masticatory muscle activity for participants' diet (K. Murakami et al., 2007). The scoring system ranged from “very soft”: 1 to “hard”: 10 (Okamoto et al., 2019; Yanagisawa et al., 1989). The foods were classified into the following 10 scores on this basis of hardness; 1: Pudding, 2: Banana, 3: Sausage, 4: Konjac, 5: Rice, 6: Mushroom, 7: Armond, 8: Radish, 9: Celery, 10: Carrot. Participants were classified into three groups according to the dietary hardness score (Group 1; 10, Group 2; 9–7, Group 3; 6–1). These group classifications were based on the previous report that measure the shear strength of foods using a V‐shape plunger (Okamoto et al., 2019). We categorized the groups equally by shear strength (Group 1: under 2.93 kgf, Group 2: under 4.38 kgf, Group 3: under 6.56 kgf).

### 2.4 Number of remaining teeth

The number of remaining teeth was counted by trained dentists, then the participants were divided into four groups based on this (Group 1: >20, Group 2: 19–10, Group 3: 9–1, Group 4: 0). Severe decay or tooth stump was not included in the remaining teeth.

The following factors were considered possible confounders influencing dietary hardness score, the number of remaining teeth, and ADL: participants' sex (men or women), smoking history (smoker or nonsmoker), dry mouth (yes or no, question of experience having a dry mouth), and mouth rinsing (possible or impossible).

### 2.5 Statistical analysis

All data are presented as mean ± standard deviation. Differences in continuous data were analyzed using Student's *t*‐test. Categorical data were analyzed using Fisher's exact test or *χ* ^2^ test. After the data were adjusted for various confounding factors such as sex, smoking, dry mouth, and mouth rinse status, we calculated odds ratios and 95% confidence intervals for dependent ADL status using logistic regression analysis. All collected data were analyzed using JMP Pro 13 (SAS Institute Inc., Cary, NC). The level of significance was set at *p* = .05.

## 3 RESULTS

The mean number of remaining teeth was 8.3 ± 9.2. The mean dietary hardness score was 8.3 ± 2.6. ADL status was identified in all participants: 80 were independent and 156 were dependent. The mean dietary hardness score was 9.4 ± 1.6 in independent participants and 7.7 ± 2.9 in dependent participants, and it was significantly different in independent than in dependent participants (*p* < .001). Individuals with high dietary hardness scores were more likely to be independent of ADL (*p* < .01; Table 1). However, no significant differences were observed in the number of remaining teeth and ADL status (*p* = .56).

**Table 1:** Characteristics of 90‐year‐old participants

	Dependent ADL		Independent ADL		*p*‐value
	*n*	(%)	*n*	(%)	
Sex					
Men	23	(17.3)	36	(45.0)	
Women	129	(82.7)	44	(55.0)	<.01
Current smoker					
Yes	22	(14.1)	29	(36.3)	
No	134	(85.9)	51	(63.8)	<.01
Dry mouth					
Yes	49	(31.4)	16	(20.0)	
No	107	(68.6)	64	(80.0)	.07
Mouth rinsing					
Possible	62	(39.7)	22	(27.5)	
Impossible	94	(60.3)	58	(72.5)	.08
Dietary hardness score					
Group 1 (10)	45	(28.9)	6	(7.5)	
Group 2 (9–7)	44	(28.2)	13	(16.3)	
Group 3 (6–1)	67	(43.0)	61	(76.3)	<.01
Number of remaining teeth					
Group 1 (≥20)	28	(18.0)	16	(20.0)	
Group 2 (19–10)	28	(18.0)	14	(17.5)	
Group 3 (9–1)	44	(28.2)	28	(35.0)	
Group 4 (0)	56	(35.9)	22	(27.5)	.56

Abbreviation: ADL, activities of daily living.

Logistic regression analysis showed that high dietary hardness score was negatively related to dependent ADL status after adjustment for possible confounding factors (Table 2). The prevalence of dependent ADL in individuals who scored low dietary hardness score (Group 3) was 4.70 times higher than those who scored a high dietary hardness score (Group 1). No significant difference in the prevalence of dependent ADL status could be found between groups divided by the number of remaining teeth.

**Table 2:** Logistic regression analysis of prevalence of dependent ADL, adjusted for various confounding factors, in relation to dietary hardness score and number of remaining teeth.

		Dependent ADL (ORs [95% CIs])		*p*‐value
Dietary hardness score			
Group 1 (10)		1		
Group 2 (9–7)		2.46 (1.18–5.13)		.02
Group 3 (6–1)		4.70 (1.80–12.29)		<.01
Number of remaining teeth				
Group 1 (≥20)		1		
Group 2 (19–10)		1.05 (0.40–2.75)		.91
Group 3 (9–1)		0.84 (0.37–1.94)		.69
Group 4 (0)		1.49 (0.64–3.48)		.36

Abbreviations: ADL, activities of daily living; CI, confidence interval; OR, odds ratio.

## 4 DISCUSSION

This study investigated the relationship between oral status (i.e., dietary hardness score and number of remaining teeth) and ADL status among 90‐year‐old individuals. We found that dietary hardness score based on subjective assessment was related to the ability to perform ADL. A previous study suggested that poor dentition may be related to the deterioration of the systemic health of aged individuals (Shimazaki et al., 2001). However, there were no significant differences in the number of remaining teeth and ADL status. These results suggest that subjective chewing assessment, but not the number of remaining teeth, may contribute to dependent ADL status in 90‐year‐old individuals. Similar results were reported by Takata, Ansai, Awano, Hamasaki et al. (2004). Therefore, only counting the number of remaining teeth may be insufficient to assess oral health status.

Several studies have reported that the results of objective and subjective evaluation of masticatory function are not strongly related (Pedroni‐Pereira et al., 2018; Takagi et al., 2017). This may be because objective measures only evaluate the crushing and mixing of food, whereas subjective measures assess older adults' perception of various chewing and swallowing processes (H.‐E. Kim & Lee, 2021). To date, questionnaires regarding daily diet have proven useful in epidemiologic surveys of masticatory function in older adults (Oka et al., 2003; Slade et al., 1996). The questionnaire used in this study to calculate dietary hardness scores could be easily answered by our study participants because the listed items are familiar to local Japanese residents (Yanagisawa et al., 1989).

Several studies have found a strong association of masticatory performance with grip strength, decreased skeletal muscle mass, and ADL status (Moriya et al., 2014; M. Murakami et al., 2015; Takata, Ansai, Awano, Sonoki et al., 2004). However, few human studies have investigated the association between dietary hardness score and ADL status. The results of the present study provide the importance of interdisciplinary work or interprofessional collaboration.

Replacing missing teeth contributes to improved mastication. Tooth loss compromises one's ability to masticate, and impaired mastication may affect the nutritional status or undermine general well‐being. A previous study demonstrated that chewing ability (not the number of teeth) and physical ability were associated in an 80‐year‐old population (Takata, Ansai, Awano, Sonoki et al., 2004). Similarly, masticatory deterioration due to tooth loss could be associated with cognitive impairment, according to both human and animal studies (E.‐K Kim et al., 2017; Oue et al., 2013). The mechanism of the association between oral function and systemic health or brain function is not fully understood. Potential explanations have shown that mastication is associated with increased cerebral blood flow, which leads to improved brain function (Kimoto et al., 2011). Interestingly, Thomson et al. reported that cognitive function early in life is important for oral health and dentition status later in life (Thomson & Barak, 2021). They used the life course approach to propose a plausible, empirically supported explanation for the association between missing teeth and poorer cognitive function in older people. According to their study, investigating the association between dietary hardness score and ADL status may start well before old age. To extend healthy life expectancy in a super‐aged society, our findings suggest that maintaining oral function is fundamental, and dental professionals should cooperate with other professionals to detect signs of frailty and reduce the need for nursing care.

Potential limitations of this study should be noted. First, we could not include multiple variables because of the small number of participants. Our surveys were performed in a rural area in Japan; hence, it was difficult to recruit more 90‐year‐old participants. More participants should be recruited in future studies to prevent sampling bias. Second, further studies are needed to assess the association between dental status and nutrition. Finally, data on some important factors related to mastication, such as temporomandibular joint disease, periodontitis, and denture status, were not included. Further studies should include these factors.

## 5 CONCLUSION

In conclusion, self‐assessed dietary hardness score, and not the number of remaining teeth, was associated with ADL status in 90‐year‐old individuals.

## CONFLICT OF INTEREST

The authors declare no conflict of interest.

## Supporting information

Supplementary information.

## Data Availability

The data that support the findings of this study are available from the corresponding author upon reasonable request.
