# Supplementary material for: Association between dietary hardness score and activities of daily living among individuals aged 90 years
Source: Clin Exp Dent Res. 2022 Jun 24;8(5):1202–6. doi: 10.1002/cre2.622 (PMC9562580; doi:10.1002/cre2.622)
Supplement: Supplementary file 1 — Supplementary information. [file CRE2-8-1202-s001.docx]

| Total estimated assisting care time (min per day) | Care level |
| --- | --- |
| t < 25 | Independent |
| 25 ≤ t < 32 | Support Required |
| 32 ≤ t < 50 | Care Level 1 |
| 50 ≤ t < 70 | Care Level 2 |
| 70 ≤ t < 90 | Care Level 3 |
| 90 ≤ t < 110 | Care Level 4 |
| 110 ≤ t | Care Level 5 |

Supplemental table 1. Definitions of care levels used for long-term care insurance eligibility qualification in Japan

Independent ADL

Dependent ADL
